# Blind image quality assessment via probabilistic latent semantic analysis

**DOI:** 10.1186/s40064-016-3400-1

**Published:** 2016-10-04

**Authors:** Xichen Yang, Quansen Sun, Tianshu Wang

**Affiliations:** School of Computer Science and Engineering, Nanjing University of Science and Technology, Xiaolingwei 200, Nanjing, China

**Keywords:** Blind image quality assessment, Probabilistic latent semantic analysis, Visual word dictionary, Distortion-affected

## Abstract

We propose a blind image quality assessment that is highly unsupervised and training free. The new method is based on the hypothesis that the effect caused by distortion can be expressed by certain latent characteristics. Combined with probabilistic latent semantic analysis, the latent characteristics can be discovered by applying a topic model over a visual word dictionary. Four distortion-affected features are extracted to form the visual words in the dictionary: (1) the block-based local histogram; (2) the block-based local mean value; (3) the mean value of contrast within a block; (4) the variance of contrast within a block. Based on the dictionary, the latent topics in the images can be discovered. The discrepancy between the frequency of the topics in an unfamiliar image and a large number of pristine images is applied to measure the image quality. Experimental results for four open databases show that the newly proposed method correlates well with human subjective judgments of diversely distorted images.

## Background

With the explosion of multimedia development, the perceptual optimization of multimedia services has gained importance. To provide end-users with the best quality experience, it is important to assess the quality of an image that may be corrupted by multiple distortions. Image quality assessment (IQA) methods can be divided into subjective assessment by humans and objective assessment by algorithms. Subjective assessment is time consuming, cumbersome, and expensive, and cannot be implemented using computer equipment. There has thus been increasing interest in developing an objective IQA that mimics subjective judg-ments.

According to the degree of information that is needed, objective IQA can be divided into three categories (Bovik 2013): (1) full-reference (FR) image assessment, (2) reduced-reference (RR) image assessment, and (3) no-reference (NR) image assessment. Considerable progress has been made for FR and RR IQA in recent years (Wang et al. 2004; Wang and Zhong-Fu 2006; Tong et al. 2009; Dumic et al. 2014; Liu et al. 2009; Wang and Rong 2011). However, reference information may be difficult to obtain in a real-time IQA process. Hence, NR IQA may be more applicable in a practical sense. NR IQA can be roughly divided into distortion-specific and universally purposed NR IQA. Distortion-specific NR IQAs are made under the assumption that the image quality is affected by one or several particular kinds of distortions, such as blockiness (Wang et al. 2000; Pan et al. 2004), rings (Liu et al. 2010), blurring (Ferzli and Karam 2009; Varadarajan and Karam 2008), and compression (Sheikh et al. 2005; Babu et al. 2007; Sazzad et al. 2008; Liang et al. 2010). The application domain of these approaches is limited as they are only suitable for the presumed distortion types. In contrast, uni-versally purposed models (Li et al. 2011; Mittal et al. 2013; Zhang et al. 2015; Moorthy and Bovik 2011; Saad et al. 2012; Mittal et al. 2012; Moorthy and Bovik 2010; Saad et al. 2010) are intended to handle multiple, possibly unknown distortions and typically involve machine learning techniques. GRNN (Li et al. 2011) deployed a generalized regression neural network combined with image perceptually relevant features to train an IQA model. DIIVINE (Moorthy and Bovik 2011) is a later extended version of BIQI (Moorthy and Bovik 2010), and both are based on a two-step framework for quality estimation. The BRISQUE (Mittal et al. 2012) model deploys a space-domain natural scene statistic (NSS) model to quantify possible losses of naturalness in an image due to the presence of distortions. However, the abovementioned algorithms require auxiliary information in the form of human opinion scores, and most are based on machine learning principles used to teach the regression model. These NR IQAs are thus only suitable for images whose distortion types have already been trained for, resulting in weak generalization capability. Collecting enough training samples of all such manifold distortion types, and then obtaining their human opinion scores, is an expensive and time-consuming procedure.

On the basis of the shortcomings mentioned above, it is meaningful to develop a highly unsupervised IQA method that requires no training on human opinion scores and does not need training samples of distortions. In reference (Mittal et al. 2012), the author proposed a blind IQA method based on latent quality factors (LQF), which conducts probabilistic latent semantic analysis (PLSA) on the NSS-based features of the image patches of the training set.

Motivated by the success of PLSA for image latent topic discovery, we propose a blind IQA based on PLSA. The framework of our method mainly contains (1) feature extraction based on grayscale fluctuation (GF) (Yang et al. 2014) analysis; (2) construction of a dictionary of visual words; (3) discovery of image latent distortion-affection topics via PLSA; and (4) measurement of image quality. The main benefits of our method are (1) that it is highly unsupervised and requires no a priori in-formation such as human opinion scores; (2) it has a distortion-affected feature section that reveals image structural information in terms of both intensity and distribution; and (3) it is not distortion-specific and effective across multiple distortion types. In this paper, we compare the performance of the proposed method with that of established methods on four open databases: LIVE2 (Sheikh et al. 2006), CSIQ (Larson and Chandler 2010), TID2008 (Ponomarenko et al. 2009) and LIVE Multiply Distorted (Jayaraman et al. 2012). The experimental results for the four open databases show that the newly proposed method accords closely with human subjective judgments of diversely distorted images.

The remainder of this paper is organized as follows. In the next section, we discuss the grayscale fluctuation analysis. In "Proposed approach" section, we introduce our method in detail. The experimental results of the proposed method are presented in "Experiments and results" section, followed by the conclusions in "Conclusion" section.

**Fig. 1:**
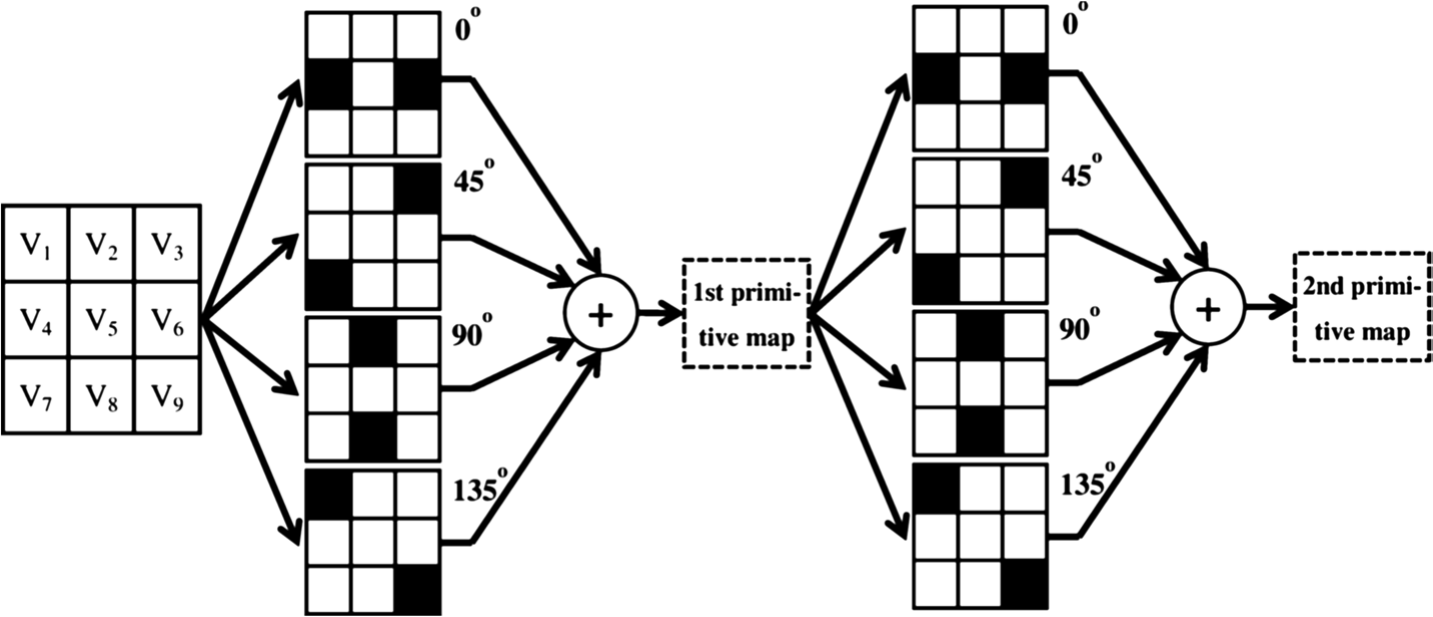
GF primitive analysis flowchart

## Grayscale fluctuation analysis

Image degradation refers to imaging that fail to fully reflect the true content of the scene, and it affects image features in terms of, for example, smoothing, sparsity and regularity. A direct effect on an image is a change to the image texture (Liu and Yang 2008; Acharya and Ray 2005). Meanwhile, the image GF reflects changes in image texture. We can thus learn the degree of image degradation by analyzing the image GF.

### Grayscale fluctuation primitive

Using the GF primitive (Yang et al. 2014), we can analyze the image GF relationships between a certain pixel and its neighbors. As shown in Fig. 1, the primitive is a $3\times 3$ square centered at $V_5$. To obtain the GF relationship between the center pixel and all its neighbors, the detection directions are set to $0^\circ$, $45^\circ$, $90^\circ$, and $135^\circ$.

**Fig. 2:**
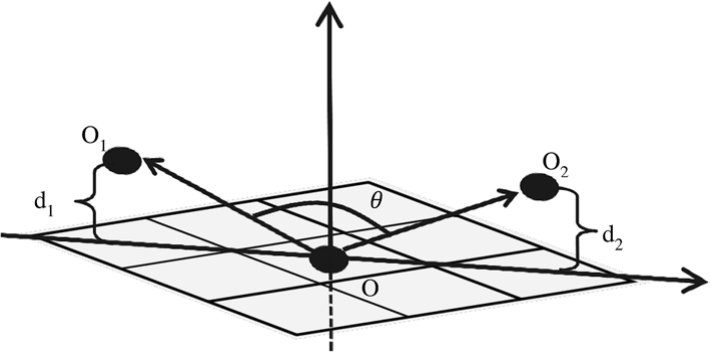
Calculation of $$Ga_x$$

The neighboring pixel grayscale vector angle (GVA) and neighboring pixel grayscale mutually exclusive value (MEV) are two variables used to represent image GF. These two variables are denoted $Ga_x$ and $Dt_x$ $\left\{ x=1, 2, 3, 4\right\}$ respectively. The method of calculating $Ga_x$ is shown in Fig. 2. The central pixel of the primitive is defined as the origin of the coordinate axes (*o*). $o_1$ and $o_2$ are the neighboring pixels in the current detection direction. $d_1$ and $d_2$ are the absolute values differences between the central pixel and its neighbors; these two variables are calculated by $d_1=\left| g\left( o\right) -g\left( o_1\right) \right|$ and $d_2=\left| g\left( o\right) -g\left( o_2\right) \right|$, where $g\left( o\right)$ and $g\left( o_i\right)$ represent the gray values of *o* and its neighbor $o_i$. The coordinates of $o_1$ and $o_2$ are denoted $\left( -1,d_1\right)$ and $\left( 1,d_2\right)$. In addition, $\theta$($\left[ 0,180^\circ \right]$) is the GVA used to reflect the GF of the center pixel. GF increases with decreasing $\theta$. $\cos \theta$ is thus used to represent $Ga_x$. $Ga_x$ is calculated according to formula (1) and ranges $\left[ -1,1\right]$.

1 $$\begin{aligned} Ga_x = \frac{d_1\times d_2-1}{\sqrt{1+{d_1}^2}\times \sqrt{1+{d_2}^2}} \end{aligned}$$

$Dt_x$ represents the change trend of GF in the current detector direction and is assigned to be 1 or $-1$, and is calculated as follows:

2 $$\begin{aligned} \left\{ \begin{array}{ll} Dt_x=1, & \quad c_1\times c_2>0,\\ Dt_x=-1, & \quad c_1\times c_2<0, \end{array}\right. \end{aligned}$$

where $c_1=g\left( o\right) -g\left( o_1\right)$ and $c_2=g\left( o\right) -g\left( o_2\right)$. The GF has a relatively big change trend when the plus-or-minus signs of $c_1$ and $c_2$ are different.

**Fig. 3:**
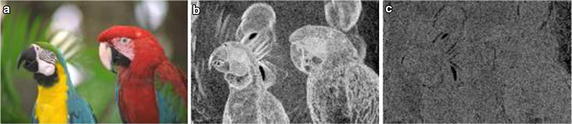
Image “parrots” and its primitive maps. **a** is the reference image. **b** is the first primitive map. **c** is the second primitive map

**Fig. 4:**
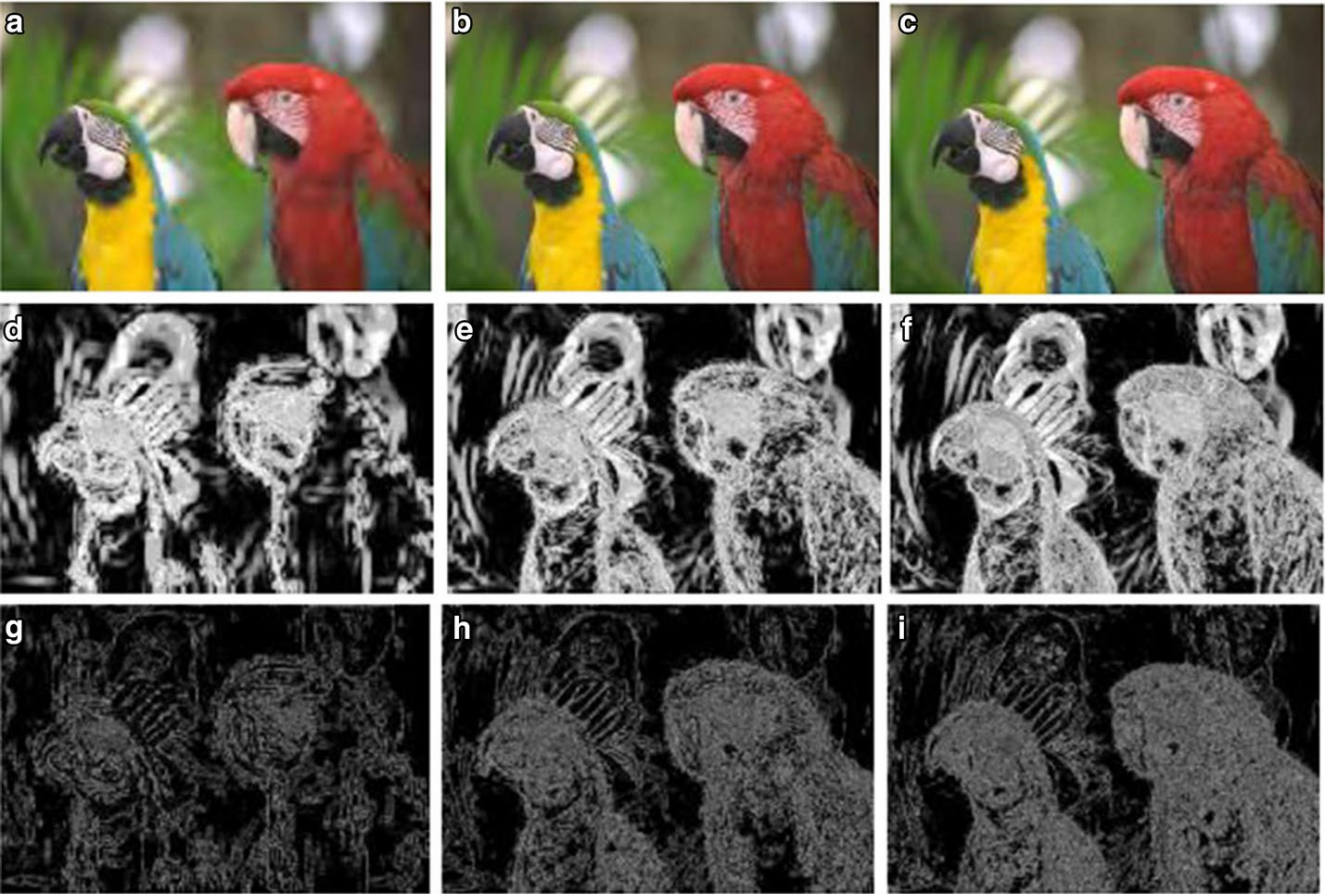
JPEG2000 distortion images and their primitive maps. **a**–**c** Are distortion images sorted in order of descending degree of distortion. **d**–**f** Are the first primitive maps of (**a**–**c**). **g**–**i** Are the second primitive maps of (**a**–**c**)

**Fig. 5:**
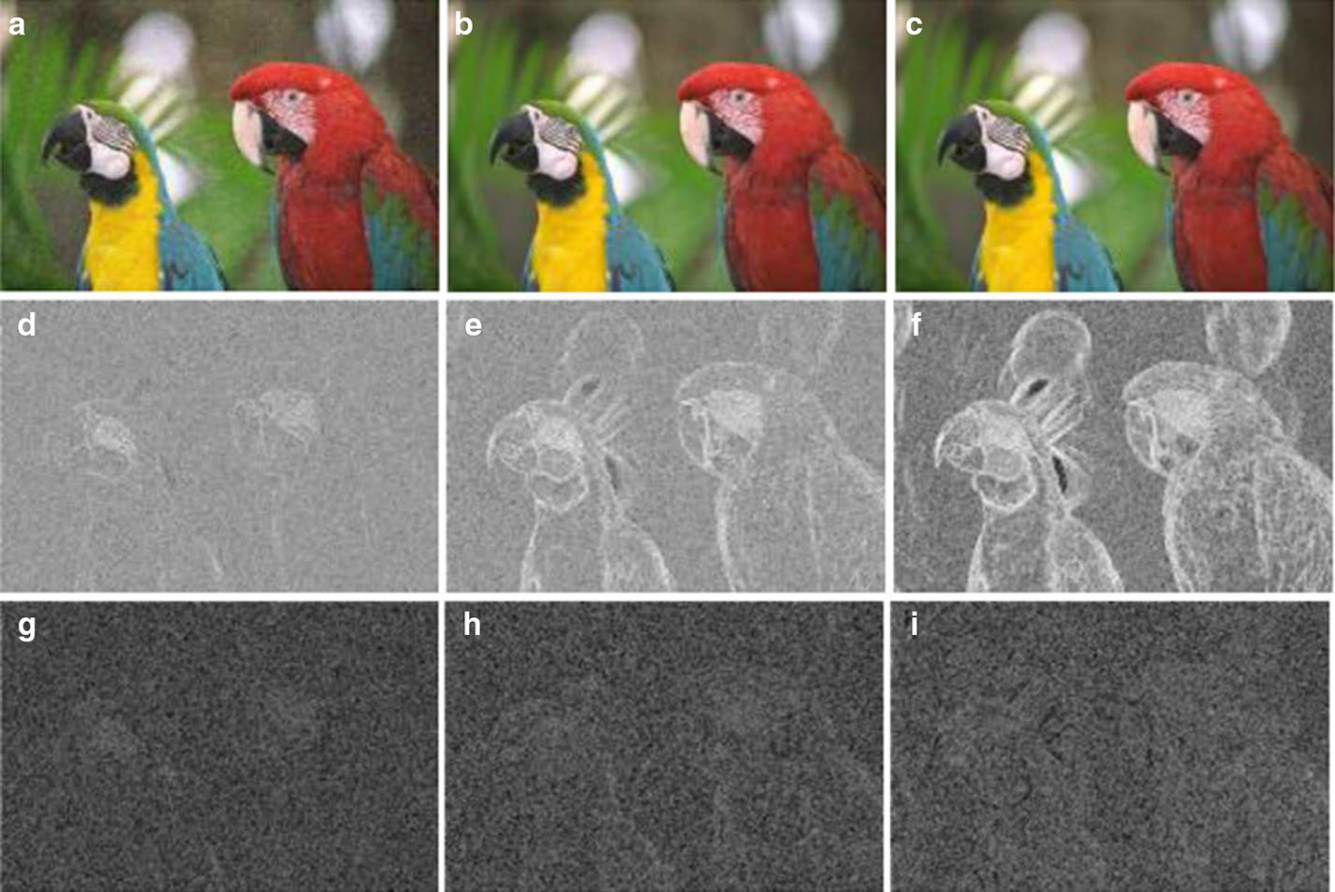
WN-distorted images and their primitive maps. **a**–**c** Are distortion images sorted in order of descending degree of distortion. **d**–**f** Are the first primitive maps of (**a**–**c**). (**g**–**i**) Are the second primitive maps of (**a**–**c**)

### Grayscale fluctuation primitive map

The GF primitive is employed to analyze an image pixel-by-pixel. The GF of the central pixel can be calculated as

3 $$\begin{aligned} \left\{ \begin{array}{ll} B_x=B_x+1, &\quad Ga_x> \cos \varphi ,\\ B_x=B_x+1, & \quad Dt_x=-1,\\ \end{array} \right. \nonumber \\ B = \sum B_x, \quad {x=1,2,3,4}, \end{aligned}$$

where $B_x$ is the GF value in one certain detection direction and *B* is the overall GF value of the central pixel. The initial values of $B_x$ and *B* are set to 0, thus, $B_x$ and *B* are both integers. In addition, $\varphi$ is the threshold of $Ga_x$. The value of *x* is $\left\{ x=1, 2, 3, 4\right\}$, thus, the range of $B_x$ and *B* are $\left[ 0,2\right]$ and $\left[ 0,8\right]$. Note that *B* is an integer; hence, there are 9 different values of *B*. By utilizing *B* to replace the value of the central pixel in the corresponding location of the image, we can obtain the GF map. The values of the pixels in the GF map are proportional to the grayscale fluctuations between the pixels and their neighbors in the corresponding location of the original image. The flowchart of the calculation procedure of GF map is shown in Fig. 1. We obtain the first GF map through the first primitive analysis. Then, by analyzing the first GF map, we obtain the second GF map.

GF map represents the relationship between pixels and their neighbors in the original image. Thus, it can reflect the degree of change in the image texture and can be used to further analyze the degree of image distortion. To demonstrate this fact, we select one reference image from the LIVE2 image database and versions of the image with different degrees of distortion. Figure 3 shows the reference image named “parrots” and its first and second primitive maps. Figure 3a is the reference image whose difference mean opinion score (DMOS) is 100, Fig. 3b is the first primitive map of (a), and Fig. 3c is the second primitive map of (b). The first primitive analysis threshold is $\varphi _1=90^\circ$ and the second primitive analysis threshold is $\varphi _2=90^\circ$. Corresponding to Figs. 3 and 4 shows JPEG2000 distortion images of the reference image sorted in descending order of the degree of distortion from (a) to (c). Using $\varphi _1$ and $\varphi _2$, we get the primitive maps, as shown in (d–i). In the same way, we obtain the primitive maps of images distorted by white noise (WN), as shown in Fig. 5. We draw the following conclusions from Figs. 3, 4 and 5. First, there is a clear connection between primitive maps and the degree of distortion. Second, the behavior of the first primitive map is more representative in visual effects. Third, using primitive maps to analyze distortion may be applicable for different distortion types. According to the above discussion, we use primitive maps to analyze image quality.

**Fig. 6:**
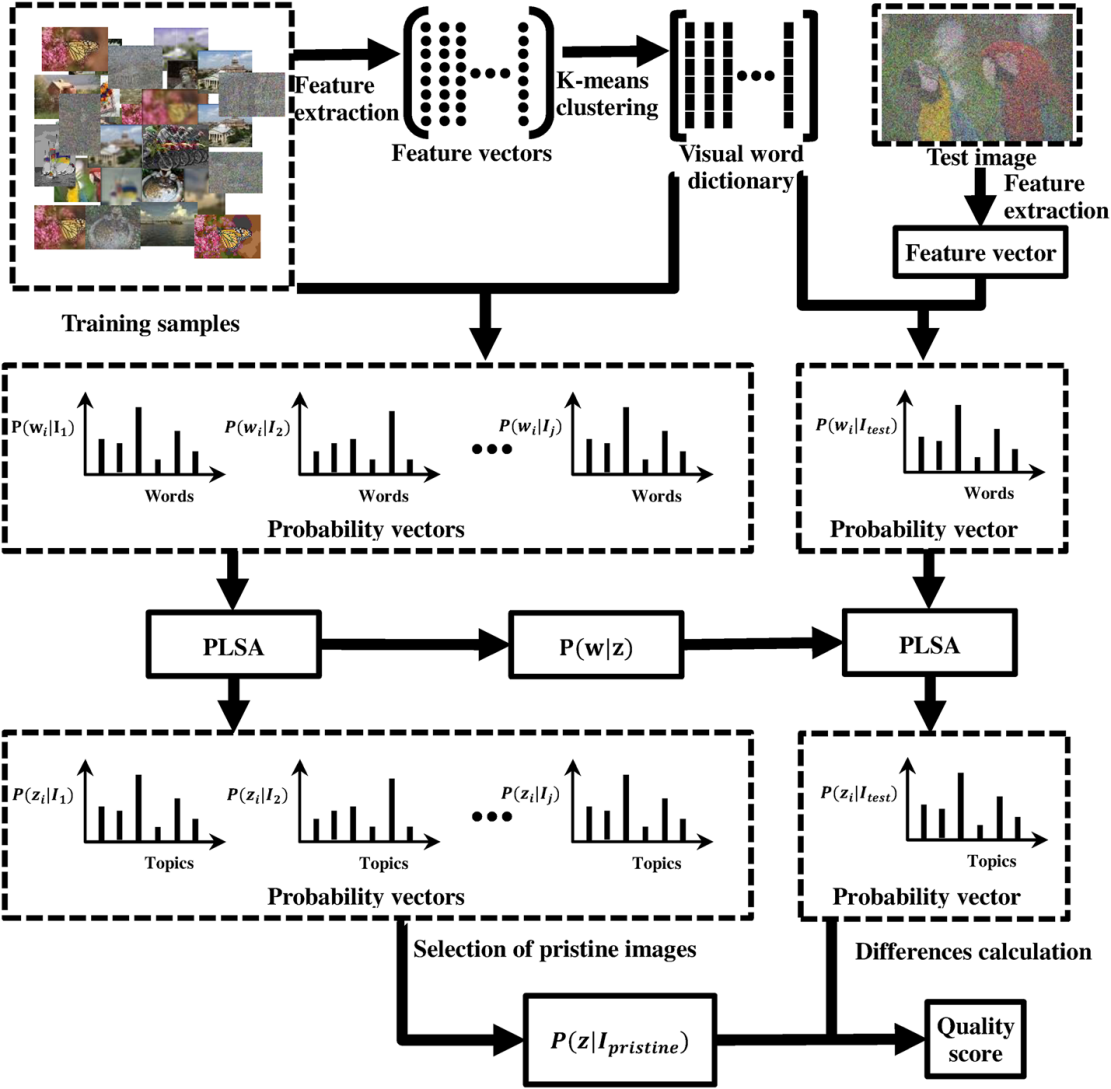
Flowchart of proposed approach

## Proposed approach

This section discusses the proposed approach in detail. The approach can be divided into four steps: (1) the GF primitive is employed to extract distortion-affected features; (2) a visual word dictionary is constructed by relying on the extracted features; (3) the PLSA algorithm is used to discover the latent topics in images; and (4) the differences between the probabilities over latent topics that are found in the test image and the pristine images are used as the measurement of image quality. The flow chart of the proposed approach is shown in Fig. 6.

### Feature extraction

**Fig. 7:**
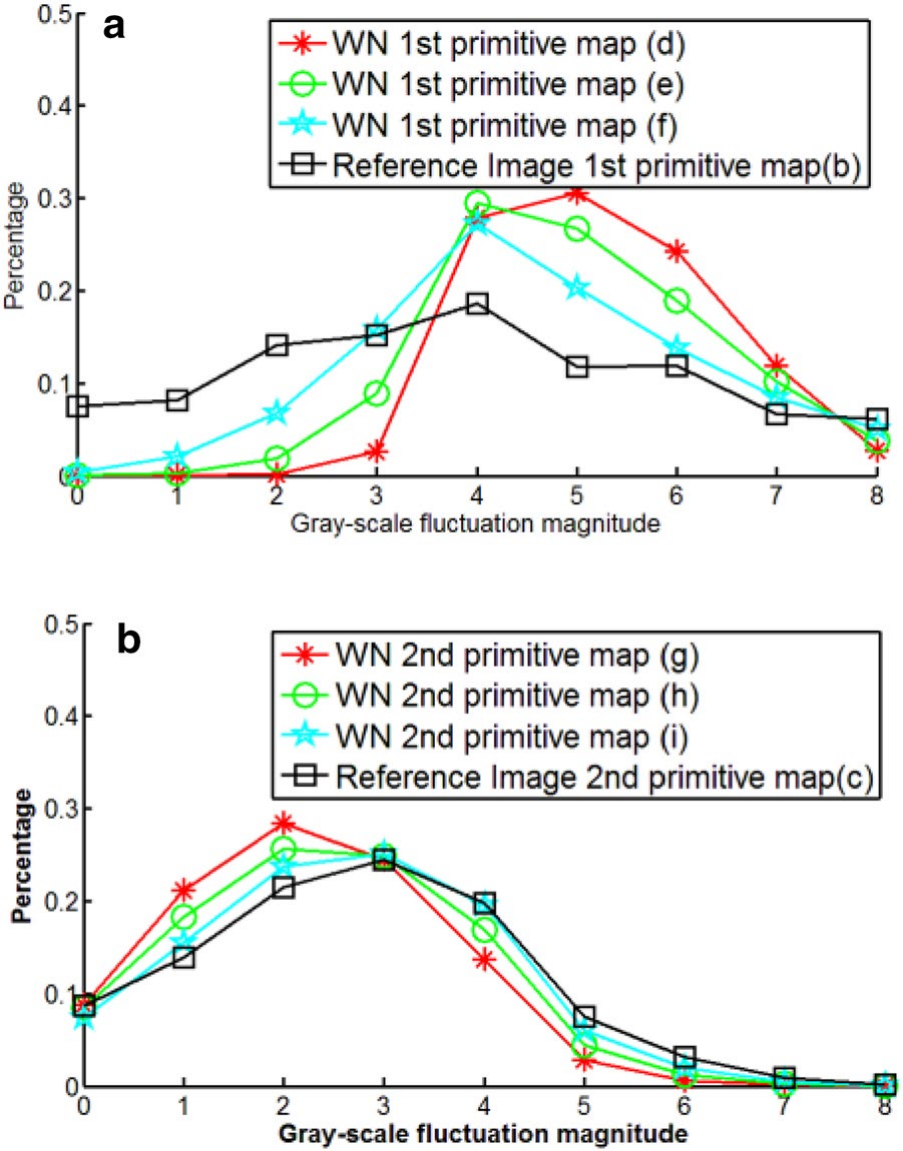
Histograms of primitive maps. **a** First primitive maps, **b** second primitive maps

It has been shown that the image GF primitive map and the degree of image distortion are closely correlated. Hence, we take the image GF primitive map as a rich descriptor of image quality. The values of pixels in the primitive map reflect the GF situation. Hence, the histogram is the most direct representation of the distribution of values in a primitive map. Figure 7 presents histograms of the first and second primitive maps of WN-distorted images. Histogram curves in Fig. 7 correspond to the first and second primitive maps shown in Fig. 5. Figure 7 provides interesting findings. First, distortions affect the distributions of the values of pixels in primitive maps. Second, the first and second primitive maps have different shapes and properties of the histogram curve. We have found that these phenomena are broadly observed in natural images not reported here. The image texture has regional characteristics. By block analyzing image primitive maps, we can learn the texture situation of the local area. We thus use the block-based local histogram of the primitive map as quality-aware features to measure image quality. Additionally, the block-based local mean-value of pixel values in the image primitive map is chosen to represent the intensity of the GF situation in different image regions. We define the block-based local mean-value as $M_b$, and the calculation formula is given as formula (4). In formula (4), the dimensions of the image block are $n\times n$, and *I*(*i*, *j*) is the value of the pixel in the designated location.

4 $$\begin{aligned} M_b=\frac{1}{n\times n}\sum _{i=1}\sum _{j=1}{I(i,j)} \end{aligned}$$

The use of contrast is an effective way of analyzing the correlation between pixels in a block. We divided the primitive map into overlapping blocks having the dimensions $n\times n$, with an overlap of ${n_x}\times {n_x}$ between neighboring blocks. In each block, the local contrast is computed over each patch of size ${n_p}\times {n_p}$. The calculation method used in this paper is the root mean square (RMS) contrast method (Peli 1990), where the RMS contrast is defined as the standard deviation of the pixel intensities, calculated as follows.

5 $$\begin{aligned} C_{patch}=\sqrt{\frac{1}{{n_p}^2}\sum _{i=1}^{n_p}\sum _{j=1}^{n_p}{(I(i,j)-\overline{I}})^2} \end{aligned}$$

Here, *I*(*i*, *j*) is the intensity at the designated location of the path, and $\overline{I}$ is the average intensity of all pixel values in the patch. Each block then has a two-dimensional feature array of size ${m_x}\times {m_x}$, ${m_x}={\lfloor {n/{n_p}}\rfloor }$. We calculate the mean and variance of the feature array, respectively denoted as $C_{mean}$ and $C_{var}$.

In summary, this paper uses the block-based local histogram, the block-based local mean value, the mean value of contrast within block and the variance of contrast within block of the primitive map as four quality-aware features with which to construct an image visual dictionary.

### Construction of a visual word dictionary

The approach we take to build the visual word dictionary is similar to that described in reference (Hartigan and Wong 1979). The visual words are formed by clustering features computed from multiple blocks across all the primitive maps in the training set, and the scale of the training set is assumed to be $N_{tr}$. Each primitive map is divided into overlapping blocks of size $n\times n$, with an overlap of ${n_x}\times {n_x}$ between neighboring blocks. According to the discussion presented in the previous section, we calculate the local histogram, $M_b$, $C_{mean}$ and $C_{var}$ over each block. The range of *B* is $\left[ 0,8\right]$ and the length of the local histogram is thus 9. Therefore, for the *i*-th primitive map in the training set sized $M_i\times N_i$, we get a two-dimensional feature matrix sized $X\times n_{b_i}$, where $X=12$ and $n_{b_i}={\lfloor {M_i/n}\rfloor }\times {\lfloor {N_i/{n}}\rfloor }$. Feature vectors from blocks across all images are gathered into a large feature matrix sized $12\times n_b$, where $n_b={\sum _{i=1}^{N_{tr}}n_{b_i}}$. Because the feature matrix is so large, the k-means clustering algorithm (Sivic 2005) with the squared Euclidean distance metric is used to reduce the dimensionality. By assigning each block to the nearest cluster center, the dimensionality of the feature matrix is reduced to $12\times n_w$. We define each cluster center as one visual word in the visual word dictionary, and use $n_w$ to denote the scale of the dictionary.

### Probabilistic latent semantic analysis

The present paper uses the probabilistic latent semantic analysis (PLSA) model of Hofmann (2001) to find certain latent characteristic differences between distorted images and pristine images. The PLSA model of Hofmann was first employed to discover latent topics embedded within a collection of text docu-ments in a corpus. In the present paper, the corpus is an assortment of pristine and distorted images. The scale of the corpus is the scale of the training set, denoted *N*. The images in the corpus can be described as an empirical distribution over visual words from the visual word dictionary. The visual words in the visual word dictionary are one-dimensional vectors. The total number of visual words contained in the dictionary is $n_w$. We suppose $I_i$ be the *i*-th image in the corpus. $I_i$ comprises $n_{w_i}$ words with the *j*-th word denoted $w_{ij}$. We then assume that there are *K* latent topics pervaded in the collection of images in the corpus, with the *k*-th topic denoted by the indicator variable $z_k$. All images in the corpus can be represented as a distribution over *K* topics, with a latent topic $z_k$ associated with each word $w_{ij}$ in the image $I_i$. The conditional probability of the word $w_{ij}$ occurring in an image $I_i$ can be obtained as follows by marginalizing over the latent topics $z_k$, with $\left\{ k = 1 \ldots K\right\}$.

6 $$\begin{aligned} P(w_{ij}\mid I_i)=\sum _{k=1}P(z_k\mid I_i)P(w_{ij}\mid z_k) \end{aligned}$$

Here, $P(z_k\mid I_i)$ is the probability of the *k*-th topic occurring in the *i*-th image and $P(w_{ij}\mid z_k)$ is the probability of the *j*-th visual word occurring in the *k*-th topic. Thus, the *k*-th topic can be represented by the $n_w$-dimensional vector $P(w_j\mid z_k)$, with $\left\{ j = 1 \ldots n_w\right\}$, and the image $I_i$ can be loaded using a *K*-dimensional vector $P(z_k\mid I_i)$, with $\left\{ k = 1 \ldots K\right\}$. Under the above assumptions, there are topics that pervade the collection of images, and their loadings for a given image can be inferred by finding the model that best explains the probability distribution of the visual words in the images. The present paper uses the expectation-maximization (EM) algorithm (Hofmann 2001) to make the maximum likelihood estimate of the model parameters. It is noted that the PLSA framework uses the “bag of words” approach as the spatial arrangement of word occurrences is not taken into account.

### Image quality inference

$P(w\mid z)$ learned from the training set that comprises both pristine and distorted images via the EM model fitting is used to infer the latent quality factors in a new image outside the training set. We denote the new image as $I_{new}$, and $P(z\mid I_{new})$ can be calculated using the fold-in heuristic described in reference (Hofmann 2001). For the new image $I_{new}$, the visual word distribution $P(w\mid I_{new})$ is first calculated. $P(z\mid I_{new})$ is then sought such that the Kull-back-Leibler divergence of the empirical visual word distribution $P(w\mid I_{new})=\sum _{k=1}^K{P(z_k\mid I_{new})P(w\mid z_k)}$ is minimized. This time EM is again employed to estimate $P(z\mid I_{new})$, but only the loadings are updated while $P(w\mid z)$ learned from the training set is held fixed. The frequency of different topics in $I_{new}$ (i.e., $P(z\mid I_{new})$) is compared with the frequency of different topics for each pristine image in the training set. We denote the frequency of different topics of each pristine image by $P(z\mid I_{p_i})$, where $I_{p_i}$ is the *i*-th pristine image in the training set. $P(z\mid I_{p_i})$ is calculated during the model fitting procedure that is carried out to learn the topic-specific word distribution $P(w\mid z)$. Referring to reference (Mittal et al. 2012), we make the comparison by computing the dot product between $P(z\mid I_{new})$ and $P(z\mid I_{p_i})$. After the dot product has been calculated across all pristine images in the training set, the average dot product is used as the indicative index of the image quality, and the quality of $I_{new}$ is denoted as $Q(I_{new})$. The calculation formula is given as formula (7).

7 $$\begin{aligned} Q(I_{new}) = \frac{1}{N_p} \sum _{i=1}^{N_p}{P(z\mid I_{new})^\prime P(z\mid I_{p_i})} \end{aligned}$$

Here, the symbol $\prime$ is the transpose operator, and $N_p$ is the number of pristine images in the training set. Owing to the linearity of the dot product, formula (7) can be written as formula (8).

8 $$\begin{aligned} Q(I_{new}) = P(z\mid I_{new})^\prime \frac{1}{N_p} \sum _{i=1}^{N_p}{P(z\mid I_{p_i})} \end{aligned}$$

Formula (7) shows that the quality assessment in this paper can be seen as a measurement of the change in the topic distribution due to distortion. The topic distribution of the undistorted image is taken as the mean value of the topic distribution of the pristine images in the training set proposed as $\frac{1}{N_p} \sum _{i=1}^{N_p}{P(z\mid I_{p_i})}$.

## Experiments and results

### Databases and metrics

To determine the performance of the newly proposed IQA method, we conduct experiments on four publicly available image databases in the IQA community, namely LIVE2 (Sheikh et al. 2006), CSIQ (Larson and Chandler 2010), TID2008 (Ponomarenko et al. 2009) and LIVE Multiply Distorted (Jayaraman et al. 2012). It is worth noting that the LIVE Multiply Distorted database includes images with multiple distortions. Images were constructed under two scenarios of multiple distortion: (1) image storage where images are first blurred and then compressed by a JPEG encoder and (2) a camera image acquisition process where images are first blurred by the narrow depth of field or another defocusing mechanism and then corrupted by white Gaussian noise to simulate sensor noise. This paper refers to the two distortion scenarios as LiveMD1 and LiveMD2, respectively.

Two commonly used performance metrics are employed to evaluate the IQA methods. The first is the Spearman rank-order correlation coefficient (SROCC), which is a measure of the prediction monotonicity of an IQA metric. The second metric is the Pearson linear correlation coefficient (LCC) for the relation between the DMOS values and objective scores after nonlinear regression. Additionally, we use the logistic function (Group 2003) to fit the results of the newly proposed method to the subjective data. On each image database, we perform a 100-fold validation experiment, and take the median value as the final result. In each run of the experiment, we randomly select the same number of reference images and their distorted versions for learning the latent quality factors, and the other reference images and their associated distorted versions are used for performance evaluation. In this way, we may minimize the interference caused by the choice of training set.

**Fig. 8:**
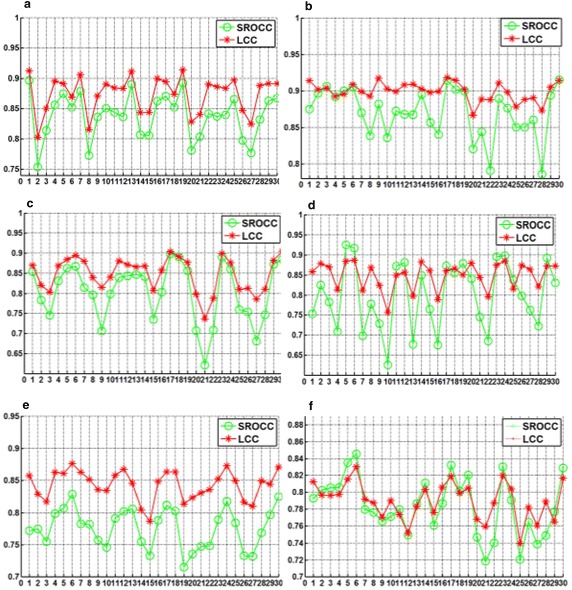
Comparative experiment for different threshold combinations on the LIVE2 database. **a** JPEG2000, **b** JPEG, **c** GBLUR, **d** WN, **e** FASTFADING, **f** OVERALL

### Performance evaluation of the grayscale vector angle threshold

$\varphi$ is an important factor of image GF analysis, and it inevitably affects IQA results. For an unseen image, the first primitive analysis threshold $\varphi _1$ is used to obtain the image first primitive map. The second primitive map can then be obtained using the second primitive analysis threshold $\varphi _2$ to analyze the first primitive map. To analyze the effect of $\varphi$ and to select the optimal threshold value, we set the groups of threshold combinations given in Table 1.

**Table 1:** Set threshold combinations

1th–6th	$\varphi _1$	$15^\circ$	$15^\circ$	$15^\circ$	$15^\circ$	$15^\circ$	$15^\circ$
	$\varphi _2$	–	$15^\circ$	$45^\circ$	$90^\circ$	$135^\circ$	$160^\circ$
7th–12th	$\varphi _1$	$45^\circ$	$45^\circ$	$45^\circ$	$45^\circ$	$45^\circ$	$45^\circ$
	$\varphi _2$	–	$15^\circ$	$45^\circ$	$90^\circ$	$135^\circ$	$160^\circ$
13th–18th	$\varphi _1$	$90^\circ$	$90^\circ$	$90^\circ$	$90^\circ$	$90^\circ$	$90^\circ$
	$\varphi _2$	–	$15^\circ$	$45^\circ$	$90^\circ$	$135^\circ$	$160^\circ$
19th–24th	$\varphi _1$	$135^\circ$	$135^\circ$	$135^\circ$	$135^\circ$	$135^\circ$	$135^\circ$
	$\varphi _2$	–	$15^\circ$	$45^\circ$	$90^\circ$	$135^\circ$	$160^\circ$
25th–30th	$\varphi _1$	$160^\circ$	$160^\circ$	$160^\circ$	$160^\circ$	$160^\circ$	$160^\circ$
	$\varphi _2$	–	$15^\circ$	$45^\circ$	$90^\circ$	$135^\circ$	$160^\circ$

We perform the comparative experiment on the LIVE2 database and the predicted quality results are illustrated in Fig. 8 for the different threshold combinations given in Table 1. In each run of the experiment, we randomly select 80 % of the reference images and their distorted versions for training, and the other 20 % reference images and their associated distorted versions for performance evaluation. We draw the following conclusions from the results presented in Fig. 8. First, the chosen threshold values affect the quality assessment results. Second, the SROCC value is more likely to be affected by threshold values. Third, each distortion type has outstanding results in that they have high SROCC and LCC values for different threshold combinations of different distortion types, but the high SROCC and LCC values of different distortion types correspond to different threshold combinations; the differences between the best and worst results are around 0.1. Hence, the prediction performance of the newly proposed IQA method has room to improve, and the ap-plicability can be improved by choosing suitable threshold combinations.

**Fig. 9:**
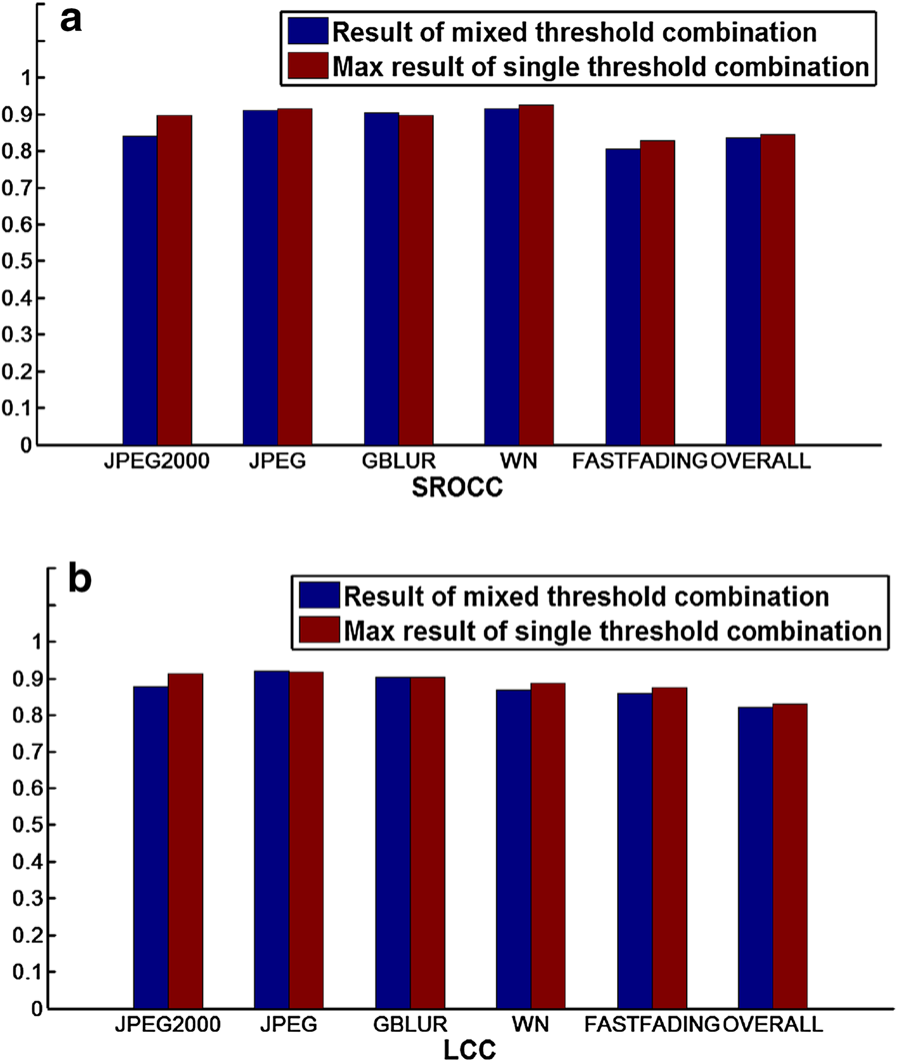
Comparison of results obtained for mixed threshold combinations and single threshold combination. **a** SROCC, **b** LCC

According to the above findings, we select threshold combinations that achieve good results for all types of distortion. The chosen threshold combinations are the 5th, 6th and 30th data in Table 1. We use the chosen threshold combinations to extract image features and then combine the features to construct the visual word dictionary. Figure 9 shows the comparison results for the mixed threshold combinations and the single threshold combinations. The result for the single threshold combinations is the optimal result among the 30 groups of threshold combinations of different distortion types. We draw the following conclusions from the results presented in Fig. 9. First, the differences in SROCC and LCC values between the mixed threshold and the best performing single group threshold are all less than 0.03 for different distortion types. Second, for most encountered distortion types in the LIVE2 database, the newly proposed IQA method delivers promising results. Third, the new assessment shows a good overall result. Therefore, by choosing suitable threshold combinations, the newly proposed method can be universally purposed without requiring auxiliary information outside the image itself, such as human opinion scores.

**Fig. 10:**
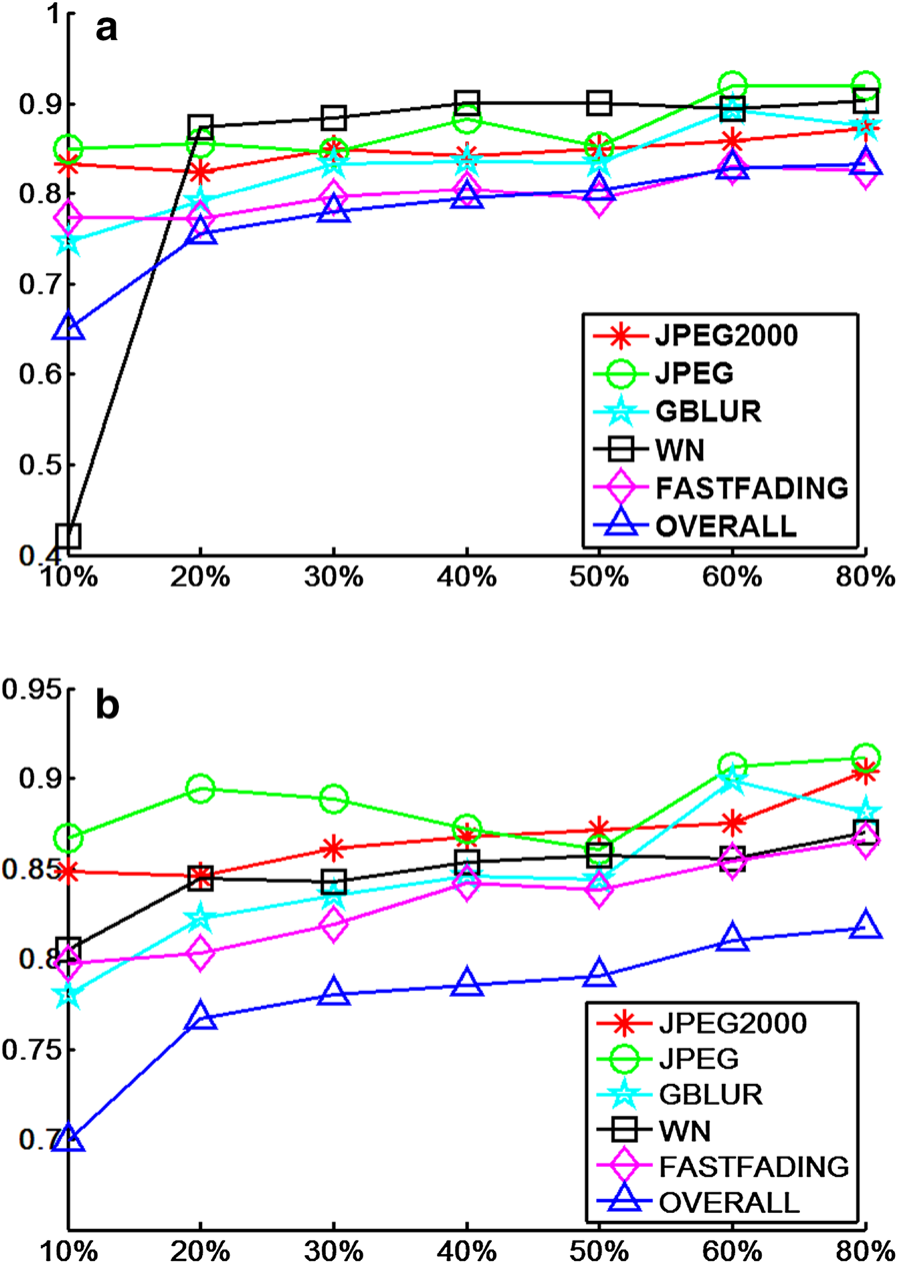
Results for different training set proportions. **a** SROCC, **b** LCC

### Performance depending on the training set proportion

Since the newly proposed method requires distorted and pristine images to teach the model, the size of the training set may affect the quality prediction results. We partition the LIVE2 database into a training set and test set. We conduct a comparative experiment for seven partition proportions; i.e., 10, 20, 30, 40, 50, 60, and 80 % of the reference images and their associated distortion images are used for the training set, while the re-maining reference images and distortion images are used for testing. The SROCC and LCC scores of different distortions are presented in Fig. 10. The figure shows that the proportion of the training set affects the performance of the newly proposed method. First, the performance of the newly proposed method for most distortion types tends to fall with a decrease in the proportion of the training set. Second, the quality assessment for the whole database gradually increases as the proportion of the training set increases. Third, the prediction results do not fluctuate greatly as the proportion ratio changes; as the proportion ratio changes from 20 to 80 %, the fluctuations of the prediction are no more than 0.05. Fourth, when the proportion ratio is 10 %, only the SROCC value of WN is under 0.65 while the SROCC and LCC values of other distortion types exceeding 0.75. The newly proposed IQA method is clearly robust against variations in the proportion ratio. In practical application, the quantity of images that need to be evaluated is tremendous when compared with the scale of the training set. The newly proposed method should be suitable for practical application as its performance is not strongly affected by variations in the proportion of the training set.

**Table 2:** Performance comparison on five databases

SROCC	NIQE	ILNIQE	LQF	PROPOSED	LCC	NIQE	ILNIQE	LQF	PROPOSED
LIVE2	*0.8316*	0.8116	0.7517	*0.8199*	LIVE2	*0.8321*	0.8311	0.7609	*0.8319*
CSIQ	0.6057	*0.7579*	0.5148	*0.6422*	CSIQ	0.6687	*0.7551*	0.5910	*0.6855*
TID2008	*0.3442*	*0.3690*	0.2083	0.3071	TID2008	0.4218	*0.4290*	0.3404	*0.4995*
LiveMD1	*0.8907*	0.7806	0.7148	*0.9006*	LiveMD1	*0.9367*	0.8060	0.8429	*0.9389*
LiveMD2	*0.7296*	0.7067	0.7208	*0.7594*	LiveMD2	*0.8168*	0.7830	0.7346	*0.8003*
Direct average performance									
SROCC	0.6804	*0.6852*	0.5821	*0.6858*	LCC	*0.7352*	0.7208	0.6540	*0.7512*
Database size based average performance									
SROCC	*0.5592*	*0.5930*	0.4502	0.5508	LCC	0.6163	*0.6293*	0.5371	*0.6541*

**Table 3:** Performance comparison on four individual distortion types

SROCC	NIQE	ILNIQE	LQF	PROPOSED	LCC	NIQE	ILNIQE	LQF	PROPOSED
LIVE2 database									
JPEG2000	*0.8851*	*0.8801*	0.8265	0.8634	JPEG2000	*0.9081*	*0.9025*	0.8529	0.8848
JPEG	0.8626	*0.8627*	0.7986	*0.8758*	JPEG	0.9086	*0.9119*	0.8583	*0.9134*
WN	*0.9673*	*0.9724*	0.8421	0.9264	WN	*0.9755*	*0.9788*	0.8756	0.8807
GBLUR	*0.9531*	0.8907	0.8699	*0.8908*	GBLUR	*0.9646*	0.9043	0.8379	*0.9086*
CSIQ database									
JPEG2000	*0.8930*	0.8140	*0.8549*	0.7958	JPEG2000	*0.9121*	*0.8127*	0.7686	0.7418
JPEG	*0.8990*	*0.9187*	0.8432	0.8211	JPEG	*0.9431*	*0.9396*	0.8527	0.8545
WN	0.8004	0.8567	*0.8634*	*0.9030*	WN	0.8031	*0.8751*	0.7903	*0.8329*
GBLUR	*0.8968*	0.8202	0.7482	*0.8369*	GBLUR	*0.9254*	*0.8514*	0.7519	0.7687
TID2008 database									
JPEG2000	*0.8970*	0.8429	0.5854	*0.8526*	JPEG2000	*0.9200*	0.8711	0.6963	*0.8736*
JPEG	*0.8774*	*0.8812*	0.7812	0.8112	JPEG	*0.9381*	*0.9459*	0.8708	0.8620
WN	*0.7186*	*0.9163*	0.6510	0.4624	WN	*0.7871*	*0.9183*	0.7123	0.6122
GBLUR	*0.8737*	*0.8338*	0.6602	0.8144	GBLUR	*0.8996*	*0.8639*	0.6847	0.8219

**Table 4:** Performance comparison on the LIVE2 database when a certain distortion type is missing

Metrics	Distortion type	JPEG2000	JPEG	GBLUR	WN	FASTFADING
SROCC	NO-JPEG2000	0.851	0.900	0.856	0.744	0.786
	NO-JPEG	0.876	0.475	0.866	0.754	0.776
	NO-WN	0.881	0.931	0.862	0.001	0.725
	NO-GBLUR	0.860	0.886	0.853	0.730	0.793
	NO-FASTFADING	0.862	0.875	0.867	0.747	0.800
LCC	NO-JPEG2000	0.867	0.917	0.884	0.833	0.869
	NO-JPEG	0.892	0.597	0.866	0.843	0.856
	NO-WN	0.895	0.926	0.874	0.324	0.817
	NO-GBLUR	0.900	0.922	0.858	0.817	0.872
	NO-FASTFADING	0.891	0.906	0.887	0.823	0.820

**Table 5:** Performance comparison on the LIVE2 database when trained on other database

Metrics	Database	OVERALL	JPEG2000	JPEG	GBLUR	WN
SROCC	TID2008	0.8094	0.8096	0.8241	0.8289	0.8061
	CSIQ	0.8212	0.825	0.7857	0.9024	0.8194
LCC	TID2008	0.8124	0.8301	0.868	0.8317	0.8399
	CSIQ	0.8355	0.8403	0.8656	0.8577	0.8269

### Performance comparison on publicly available image databases

We compare the newly proposed IQA method with three blind IQA methods: Natural Image Quality Evaluator (NIQE) Mittal et al. (2013), Integrated Local NIQE (ILNIQE) Zhang et al. (2015), and LQF Mittal et al. (2012). In the proposed method, the number of visual words is 400 and the topic number is set to be 4. For each database, 80 % of the reference images and corresponding distorted images are selected to constitute the training set, and the remaining images constitute the test set. We repeat the train-test procedure 100 times on each database. Instead of using their own training set, in each train-test procedure we use the reference images in the randomly partitioned training set to train the model for NIQE and ILNIQE for fairness. Table 2 lists the overall performances of the IQA methods on each database. Meanwhile, the average values of SROCC and LCC over four databases are illustrated to evaluate the overall performance of the IQA methods. The average values of SROCC and LCC are calculated in two cases. In the first case, the performance metrics are directly averaged across the four databases. In the second case, different weights are given to different databases according to the number of distorted images contained in each database, i.e., 779 for LIVE2, 866 for CSIQ, 1700 for TID2008, 225 for LiveMD1 and 225 for LiveMD2. Table 3 compares the performances of the IQA methods on individual distortion types. For LIVE2, TID2008 and CSIQ, we choose four distortion types: JPEG2000 compression (JPEG2000), JPEG compression (JPEG), WN and Gaussian blur (GBLUR), since these four distortion types are the only distortion types that are included in all three databases. In Tables 2 and 3 the best two results are highlighted in italic.

We draw the following conclusions from Tables 2 and 3. First, the new IQA method correlates well with human opinion scores over all four publicly available image databases; the overall results of the proposed method are in the best two results except for the SROCC on TID2008. Second, in the first average case the proposed method performs better than its competitors. In the second average case, the proposed method performs better than its competitors in LCC. Meanwhile, in this case the SROCC average performance of the proposed method is only 0.0422 worse than the ILNIQE. Third, the SROCC values of the proposed method are in the best two results for JPEG and GBLUR on LIVE2, WN and GBLUR on CSIQ, and JEPG2000 on TID2008. The LCC values of the proposed method are in the best two values for JPEG and GBLUR on LIVE2, WN on CSIQ, and JPEG2000 on TID2008. Even in other circumstances where the prediction results are not in the best two, the results of the proposed method maintain competitiveness. Fourth, the newly proposed method outperforms LQF for almost all distortion types. Hence, the features we choose for the construction of the visual word dictionary are more quality aware. Fifth, when aimed at multiple distortion types, the newly proposed method is adaptable and has an obvious advantage; the SROCC and LCC metrics for the proposed method are among the best two results. Additionally, the two metrics are approximately 0.9 in the comparison experiments conducted on the LiveMD1 database.

On the basis of the above discussion, the proposed method is promising for the following reasons. First, the performance of the proposed method is competitive with the the state of the art IQA methods: NIQE and ILNIQE. Second, unavoidable problems must be considered; i.e., the high expense of collecting human opinion scores, the difficulty of obtaining reference images in practical applications, and most distortions in real life being multiple distortions. The proposed method does not need human opinion scores for training, and performs well in predicting the quality of images which contain multiple distortions. Third, the image is a direct expression of information, and the decay of image quality can be modeled by latent semantics associated with an information expression. Therefore, by choosing appropriate quality-aware or information-aware features to analyze image latent semantics, we may find an effective way of predicting image quality.

### Sensitivity to the training set

To measure the robustness of the proposed method, we perform comparison experiments on the LIVE2 database in two cases. In the first case, for each set of experiments, we construct the training set for all but one distortion type in the LIVE2 database. In the second case, the proposed method is trained on TID2008 and CSIQ, then tested on LIVE2. The results are shown in Tables 4 and 5.

It is seen in Tables 4 and 5 that the newly proposed assessment method performs well in terms of correlating with human opinions; the SROCC and LCC metrics exceed 0.8 for most distortion types. This demonstrates the good robustness of the proposed method. In Table 4, only when WN distortion is removed from the training set is the quality of WN-affected images not correctly predicted. One possible reason is that the characterization model of WN is different from that of the other distortion types. In future work, we may need to investigate how to develop a similarity characterization topic model of different distortion types, and decrease the sensitivity to the training distortion type.

## Conclusion

We proposed a blind quality assessment that is highly unsupervised. PLSA is used to discover latent quality semantics in a set of pristine and distorted images. The feature extraction of the newly proposed method is based on image GF analysis. The quality-aware features are taken from the obtained GF primitive map. We then use the features to construct a visual word dictionary. By using PLSA algorithm and the visual word dictionary, we discover the latent characteristics from pristine and distorted images in the training set and construct a topic model. The discrepancy of topic distribution between an unseen image and a set of pristine images is used to measure image quality. It is worth noting that the new method removes the effects of human opinion scores. Our future work will focus on gaining more distortion-affected features that are suitable for image topic model construction. According to the experimental results, the newly proposed method is a little sensitive to the distortion types in the training set. We may need to investigate the interplay between the distortion types and the image topics. In this way, we may obtain a robust model with which to infer image quality.
